# A global dataset of fossil fungi records from the Cenozoic

**DOI:** 10.1038/s41597-025-04553-4

**Published:** 2025-02-21

**Authors:** Emily Hodgson, Jessica McCoy, Kerry Webber, Noelia Nuñez Otaño, Jennifer O’Keefe, Matthew Pound

**Affiliations:** 1 Department of Geography and Environmental Sciences, Northumbria University, Newcastle upon Tyne, NE1 8ST UK; 2 Laboratorio de Geología de Llanuras, Facultad de Ciencia y Tecnología, Universidad Autónoma de Entre Ríos, Sede Diamante; CICYTTP (CONICET- Prov. ER- UADER), Diamante, Argentina; 3 Department of Engineering Sciences, Morehead State University, Morehead, KY 40351 USA

**Keywords:** Palaeoecology, Palaeoclimate, Biodiversity, Palaeontology, Taxonomy

## Abstract

There is a substantial geologic record of microfossils belonging to the fungal kingdom, however there is a need for a curation of these fossil fungi records which is up-to-date, convenient, and manipulable. Here we present MyCeno (Mycology of the Cenozoic) 2.0: a dataset containing more than 3,000 records of fossil fungi from the Cenozoic era (66 million years ago to present) and from more than 200 locations around the world. These records represent a variety of fungal body parts, but most frequently fungal spores and spore-bearing structures. Detailed information about the locations, age estimations, geology, nomenclature, and taxonomy has been collected for every record. This dataset is an access point for people wanting to utilise fossil fungal records from the Cenozoic. It can be used for understanding fungal evolution, reconstructing past environments, and studying the impacts of climate change on biodiversity during the Cenozoic.

## Background & Summary

The Cenozoic (the last 66 million years) is routinely studied to understand climate change and its impact on biota^1,2^. There is now considerable understanding of global vegetation distributions throughout the Cenozoic^3,4^. However, little attention has been paid to fungi, despite the diversity and ecological essentiality of this ancient kingdom, which is ubiquitous in all environments on Earth. Fungi evolved more than one billion years ago, have an estimated 2.5 million species, and uphold ecosystems with vital responsibilities including decomposition, nutrient mobilisation and transport, and their mutualistic symbiotic relationships with most plants^5,6^. The fossil record of fungi is surprisingly extensive; soft-bodied fungal forms like mushrooms do not generally preserve well but such fossils do exist, and there is a vast quantity of fossil spores (reproductive cells), hyphae (filamentous non-reproductive structures) and epiphyllous fungi (fungi attached to the surface of plants) which have fossilised well^7^. The Fungi in a Warmer World (FiaWW) project has been generating new fossil fungal records, compiling together pre-existing records, and showing the applications of fossil fungi to palaeoclimates and palaeoecology^8,9^.

Scientific research requiring fossil fungi data is hindered and decelerated by the inaccessibility of records. A comprehensive record of palynomorph publications that was maintained until the 2010s, including fossil fungal spores, can be found in the John Williams Index of Palaeopalynology (a physical card catalogue in the Natural History Museum, London), however this resource is not currently available digitally online^10^. Other previous efforts to collate records have been country-specific^11^ or, as for the Kalgutkar and Jansonius database of fossil fungi, whilst an admirably user-friendly search engine, it presents records described up to the year 2000 and has not had age or taxonomic information updated since 2014^12^.

**Fig. 1:**
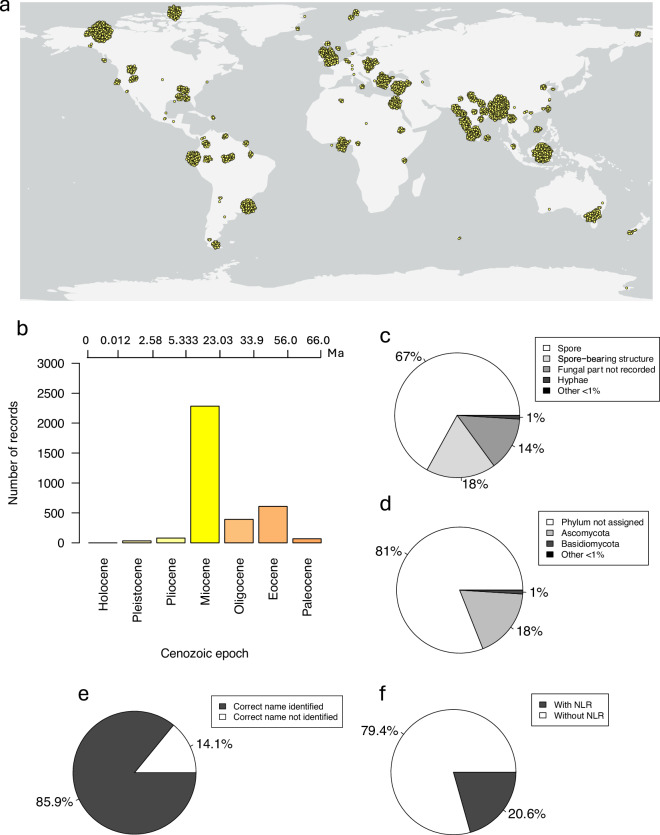
Summary of MyCeno 2.0. (**a**) Locations of records. Overlapping marker points dispersed 5 US Survey miles apart. Map created using ArcGIS Pro (Service layer credits: Esri, FAO, NOAA, USGS)^21,22^. (**b**) Number of fossil records in the dataset from each Cenozoic epoch, using the average of the maximum and minimum age estimates of each fossil record. Bars coloured according to epoch colours in the Geological Society of America’s Geologic Time Scale v. 6.0^23^. (**c**) Proportions of fungal body parts represented. (**d**) Proportions of phyla represented. (**e**) Proportion of records with and without nearest living relatives (NLRs) identified. (**f**) Proportion of records with and without correct scientific names identified.

Here we present an up-to-date and accessible global dataset of 3,484 fossil fungi records: MyCeno (Mycology of the Cenozoic) 2.0. This dataset has a wide geographical distribution, with records from 44 countries and all seven continents (Fig. 1a). The dataset includes fossils with age estimates ranging across the Cenozoic and is particularly comprehensive in its coverage of Miocene-dated fungal fossils (Fig. 1b). At least 67% of records are spores and 18% are spore-bearing structures (Fig. 1c). Detailed information about location, geology, and taxonomy has been recorded, facilitating more ways to search and analyse the data. This new synthesis has been through technical validation to update nomenclature, classification and dating, and provides a qualitative grade of dating certainty for each record. 19% of records were able to be assigned to a phylum, with 18% belonging to Ascomycota, and 1% to Basidiomycota (Fig. 1d). Nearest living relatives have been identified for 20.6% of records (Fig. 1e), and correct scientific names have been found for 85.9% of records (Fig. 1f). All record sources have citations, and 57.7% of records have an associated digital object identifier (DOI). We have provided the dataset as a comma-separated values (CSV) file, making it easy to search and manipulate using code or within Excel. This dataset can be used for understanding the distribution of fungal taxa in time and space, interpreting the evolutionary histories of fungi, and provides evidence for past environments and ancient fungal-associated biota^9,13,14^.

## Methods

### Data acquisition

**Fig. 2:**
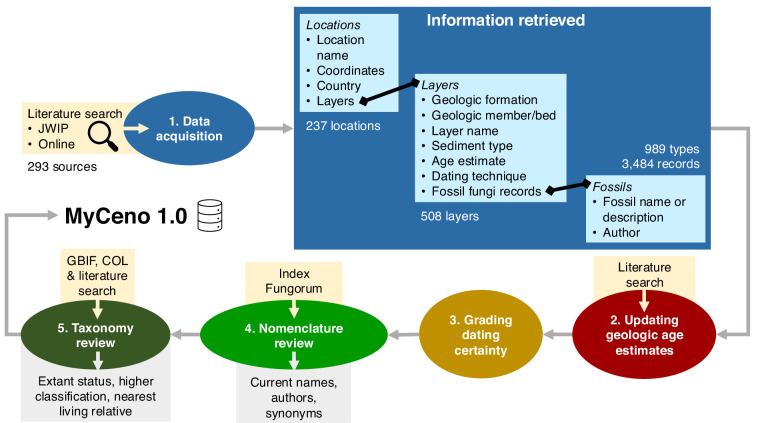
Methodology flowchart showing the synthesis of fossil fungi data into the MyCeno 2.0 dataset. Whilst information was extracted verbatim from online literature and literature from the John Williams Index of Palaeopalynology (JWIP) in step 1, steps 2 to 5 involved reviewing and updating of the geologic age, fungal nomenclature, and taxonomy using secondary literature, Index Fungorum, the Catalogue of Life (COL), and the Global Biodiversity Information Facility (GBIF).

Records of Cenozoic fossil fungi were acquired from both online peer-reviewed literature and literature from the John Williams Index of Palaeopalynology (JWIP) in the Natural History Museum, London. Miocene-dated fossil records were prioritised for transcription. From these sources, information about the location, geologic layers, and fossils present were recorded (Fig. 2). Location coordinates were either transcribed directly from the literature or if not available, were estimated using the description of the location in the source. In the latter case, this estimation was flagged in the ‘Notes’ column of the dataset.

### Updating age estimation and grading dating uncertainty

Age estimations were reviewed and updated, where possible, for obsolete or wide-ranging estimations. Newer scientific literature was consulted to check whether formations had had their ages updated. These updates are flagged under ‘Notes’, and the secondary literature sources which informed the new age estimates are referenced under the ‘Supporting_literature_IDs’ column of the dataset.

The level of dating uncertainty was determined for all records using a qualitative grading system, similar to Pound & Salzmann^15^, to be able to compare reliability of dating techniques across the dataset^15^ (Table 1). A grade of 1 indicates a very reliable age estimation based on robust dating evidence including radiometric or isotopic dating. Grades 2–4 were given if magnetostratigraphy or biostratigraphy dating techniques were used (4 for a wide numerical range and limited evidence, 2 for a narrow numerical range and multiple taxa used for biostratigraphy). Grade 5 was given if there were vague or no dating techniques mentioned in the source, or just lithostratigraphy was used. Dating techniques and dating certainty grades were based on the new updated age estimates from step 2 of the methodology workflow (Fig. 2).

**Table 1:** Qualitative grading of geologic dating techniques in MyCeno.

Grade	Age certainty	Example geologic dating techniques	% of records in this grade
1	Very high	Radiometric or isotopic dating	3.0
2	High	Biostratigraphy or magnetostratigraphy (narrow numerical range)	19.6
3	Moderate	Biostratigraphy or magnetostratigraphy (moderate numerical range)	12.1
4	Low	Biostratigraphy or magnetostratigraphy (wide numerical range)	13.3
5	Very low	Lithostratigraphy	51.9

### Nomenclatural and taxonomic review

Correct scientific names, synonyms, and higher taxonomic classifications for the fossils were determined using the Index Fungorum and Catalogue of Life databases^16,17^. On a few occasions, when names were not in the IF database, The Kalgutkar and Jansonius Database of Fossil Fungi was consulted^12^. For 15 fossil types (491 fossil records; 14.1% of records), no correct name was found for the fossil names given in sources. This includes those with descriptive titles only (e.g. hyphae, fungal spores, fungal remains etc.). For these kinds of fossils although ‘Correct_name’, ‘Correct_name_author’, ‘Is_genus_extant’, and ‘Higher_classification’ entries were left empty, recording their occurrence provides direction for future re-examination and re-collection efforts.

To determine whether a fossil genus is extant, the Global Biodiversity Information Facility (GBIF) and scientific literature were consulted^18^. GBIF was used to check for images of occurrences and whether the occurrences are annotated as fossil or living specimens. Scientific literature was consulted to see if living occurrences might be missing from GBIF.

If the fossil taxon is considered extant, this taxon was used for the nearest living relative (NLR) of the fossil. Otherwise, the next above taxonomic classification was checked for whether that was extant or not. The lowest possible extant taxonomic classification was used as the NLR for a given fossil taxon. NLRs were never assigned based on speculative comments of morphological affinity between fossil and modern fungi taxa.

## Data Records

MyCeno version 2.0 is available at 10.5281/zenodo.10792807 as three comma-separated values (CSV) files containing the records of fossil fungi (MyCeno_records.csv), citations for all dataset sources (MyCeno_citations.csv), and column information (MyCeno_column_info.csv)^19^.

## Technical Validation

Steps 2–4 of the methodology workflow, outlined in Fig. 2, are all steps to review the quality of the metadata and make updates where necessary. Age estimates were updated for 14 locations, improving the age estimations for 84 fossil records. The dating uncertainty grade provides a qualitative measure of how certain we can be of the age estimation for each fossil. Nomenclature was checked and updated for all fossil records, presented in the ‘Correct_name’ and ‘Correct_name_author’ columns, with records kept of the original fossil description under the ‘Fossil_fungus’ and ‘Author’ columns.

## Usage Notes

### Column information

The columns of the dataset are explained in the column information table (Myceno_column_info.csv) at 10.5281/zenodo.10792807. Some additional information is provided below to explain general concepts and define specialised terminology from the dataset:

- ‘ID_location’, ‘ID_layer’, ‘ID_fungus’, and ‘ID_record’ provide numeric identifiers for the different locations, layers, types of fungi, and individual records, respectively. This enables clear differentiation in the case that names might be repeated across different elements.
- ‘Higher_classification’ provides the closest higher taxonomic classification that the identified organism belongs to, from family-level upwards. For example, the higher classification for *Tetraploa aristata* is the family *Tetraplosphaeriaceae*, whereas the higher classification for *Pesavis tagluensis* is Ascomycota, because this species has not yet been placed at a lower taxonomic classification than phylum level.
- ‘Dating_technique’ contains the following categories: Biostratigraphy; Isotopic dating; Lithostratigraphy; Magnetostratigraphy; Radiometric dating. Any further detail available is written adjacent in brackets.
- ‘Sediment’ contains the following categorical variables: Amber; Brown coal or lignite; Chert; Clay; Claystone; Conglomerate; Diamictite; Diverse; Goethite; Kaolinite; Lacustrine, black clay; Limestone; Marl; Mudstone; No data; Paleosol; Sandstone; Schist; Shale; Silt; Siltstone; Tuff; Wood.
- ‘Fossil_type’ describes the anatomical fungal body part that has been fossilised. Categories and their definitions are:
  - Spore = sexual or asexual fungal unit of reproduction made up of one to several cells.
  - Spore-bearing structure = reproductive structure that produces spores, such as a basidiocarp (spore-bearing structure of phylum Basidiomycota), conidiophore (asexual spore-bearing structure), perithecium (round or flask-shaped spore-bearing structure), or thyriothecium (flattened spore-bearing structure on the surface of living or dead leaves or stems of plants).
  - Hyphae = long filamentous fungal structure.
  - Lichen thallus = main body of a lichen (symbiotic organism made up of at least a fungus and an alga or cyanobacterium).
  - Other.

### Limitations of the dataset

Users of the dataset should be aware that nomenclature and taxonomy are continually changing, especially for the fungal kingdom^20^. The nomenclature and taxonomy in MyCeno 2.0 were established between September 2023 and July 2024 using Index Fungorum and the Catalogue of Life, however there will very likely be future updates from taxonomists that will not be actively changed in version 2.0. Furthermore, whilst Index Fungorum is the most up to date and actively maintained database for checking nomenclature of fungal taxa, and the Catalogue of Life is similarly thoroughly maintained in its classifications, they are not always completely current. Some of MyCeno’s “correct names” and taxonomic information will be wrong if incorrect on Index Fungorum or the Catalogue of Life too.

Similarly, the dating of geologic formations may change as new research is conducted. Therefore, fossil age estimates may become inaccurate in the future and in need of revision.

MyCeno 2.0 covers Miocene records well, but other epochs are incomplete. Future versions will aim for a fully comprehensive collection of records.

There may also be minor spelling mistakes and errors due to the quantity of manual transcription from the literature that was involved in making this dataset.

## Supplementary information

Table 1

## Data Availability

No custom code has been used to create this dataset.

## References

[1] Steinthorsdottir, M. *et al*. The Miocene: The future of the past. *Palaeoceanogr. Palaeoclimatol.* **36**, e2020PA004037 (2021).

[2] Guo, W.-Y. *et al*. Climate change and land use threaten global hotspots of phylogenetic endemism for trees. *Nat. Commun.* **14**, 6950 (2023). 37907453 10.1038/s41467-023-42671-y PMC10618213

[3] Pound, M. J., Haywood, A. M., Salzmann, U. & Riding, J. B. Global vegetation dynamics and latitudinal temperature gradients during the mid to late Miocene (15.97–5.33Ma). *Earth Sci. Rev.* **112**, 1–22 (2012).

[4] Korasidis, V. A., Wing, S. L., Shields, C. A. & Kiehl, J. T. Global changes in terrestrial vegetation and continental climate during the Paleocene‐Eocene Thermal Maximum. *Palaeoceanogr. Palaeoclimatol.* **37**, e2021PA004325 (2022).

[5] Berbee, M. L., James, T. Y. & Strullu-Derrien, C. Early diverging fungi: diversity and impact at the dawn of terrestrial Life. *Annu. Rev. Microbiol.* **71**, 41–60 (2017). 28525299 10.1146/annurev-micro-030117-020324

[6] Niskanen, T. *et al*. Pushing the frontiers of biodiversity research: unveiling the global diversity, distribution, and conservation of fungi. *Annu. Rev. Environ. Resour.* **48**, 149–176 (2023).

[7] Taylor, T. N., Krings, M. & Taylor, E. L. *Fossil Fungi*. (Elsevier Science, 2014).

[8] Romero, I. C. *et al*. First record of fungal diversity in the tropical and warm-temperate middle Miocene Climate Optimum forests of Eurasia. *Frontiers in Forests and Global Change* **4** (2021).

[9] Pound, M. J. *et al*. The fungal ecology of the Brassington Formation (middle Miocene) of Derbyshire, United Kingdom, and a new method for palaeoclimate reconstruction. *Front. Ecol. Evol*. **10** (2022).

[10] Riding, J. B., Pound, M. J., Hill, T. C. B., Stukins, S. & Feist-Burkhardt, S. The John Williams Index of Palaeopalynology. *Palynology* **36**, 224–233 (2012).

[11] Saxena, R. K. & Tripathi, S. K. M. Indian fossil fungi. *Journal of Palaeosciences* **60**, 1–208 (2011).

[12] Berbee, M., Le Renard, L. & Carmean, D. Online access to the Kalgutkar and Jansonius database of fossil fungi. *Palynology* **39**, 103–109 (2015).

[13] White, J. M. & Jessop, C. M. Population-based analysis and graphic interpretation of fossil palynomorph records from Palynodata: taxonomic and biostratigraphic implications. *Palaeogeogr. Palaeoclimatol. Palaeoecol.* **180**, 129–146 (2002).

[14] Marret, F., O’Keefe, J., Osterloff, P., Pound, M. & Shumilovskikh, L. Applications of Non-Pollen Palynomorphs: from Palaeoenvironmental Reconstructions to Biostratigraphy. *Geological Society, London, Special Publications* **511** (2021).

[15] Pound, M. J. & Salzmann, U. Heterogeneity in global vegetation and terrestrial climate change during the late Eocene to early Oligocene transition. *Sci. Rep.* **7**, 43386 (2017). 28233862 10.1038/srep43386 PMC5324063

[16] Royal Botanic Gardens, Kew, Landcare Research-NZ & contributors. *Index Fungorum*. https://www.indexfungorum.org (2023).

[17] Bánki, O. *et al*. Catalogue of Life. 10.48580/dgjc7 (2024).

[18] Global Biodiversity Information Facility (GBIF) Home Page. https://www.gbif.org/ (2023).

[19] Hodgson, E., McCoy, J., Webber, K. & Pound, M. MyCeno (2.0). *Zenodo*10.5281/zenodo.14037140 (2024).

[20] Naranjo‐Ortiz, M. A. & Gabaldón, T. Fungal evolution: diversity, taxonomy and phylogeny of the Fungi. *Biological Reviews* **94**, 2101–2137 (2019). 31659870 10.1111/brv.12550 PMC6899921

[21] Esri. Light Gray Canvas. https://www.arcgis.com/home/item.html?id=979c6cc89af9449cbeb5342a439c6a76> (2024).

[22] Esri. ArcGIS Pro. https://www.esri.com/en-us/arcgis/products/arcgis-pro/overview (2023).

[23] Walker, J. D. *GSA Geologic Time Scale v. 6.0.*10.1130/2022.CTS006C (2022).

